# Comparing Insect Predation by Birds and Insects in an Apple Orchard and Neighboring Unmanaged Habitat: Implications for Ecosystem Services

**DOI:** 10.3390/ani13111785

**Published:** 2023-05-27

**Authors:** Moshe Nagari, Motti Charter

**Affiliations:** 1 Shamir Research Institute, University of Haifa, Katzrin 1290000, Israel; 2 Department of Geography and Environmental Sciences, University of Haifa, Mount Carmel, Haifa 3498838, Israel

**Keywords:** eco-system services, great tits *Parus major*, beneficial insects, pests, pesticides

## Abstract

**Simple Summary:**

The study aimed to understand the differences in predation of insect pests between managed apple orchards and surrounding habitats. The researchers used mealworm (*Tenebrio molitor*) pupae as a proxy for coddling moth (*Cydia pomonella*) pupae and placed them in 42 stations within the orchard and 42 stations in a Eucalyptus stand. Half of the stations were covered with nets to prevent bird predation, while the other half were accessible to birds and insects. The researchers recorded the predation of the pupae and analyzed the videos to determine which species were responsible for the predation. They found that in net-free stations, the predation rate inside the orchard was higher than in the Eucalyptus stand, and the video analysis confirmed that birds were responsible for most of the predation. In netted stations, the predation rate was higher in the Eucalyptus trees, and most of these predations were carried out by ants. The results suggest that the orchard environment negatively affects insect activity as the distance into the orchard increases, specifically predatory ants and that bird predation of insect pests inside the orchard may be more intense than in the surrounding unmanaged habitat. This study highlights the importance of considering the impact of insecticides on the activity of beneficial insect predators in agriculture and suggests that birds may play an important role in controlling insect populations in the orchard.

**Abstract:**

Preserving ecosystem services, such as natural enemies that can provide pest control, can positively impact crops without compromising agricultural yield. Even though controlling pests by natural enemies has been suggested to reduce pests in agriculture, growers continue using conventional pesticides that kill beneficial predators. Here we studied whether the predation of avian and insect-beneficial predators varies in an apple orchard with conventional insecticide use compared to a bordering tree stand without insecticides. We studied the predation rates of mealworm pupae as a proxy to coddling moth pupae at 42 stations in both an apple orchard and a Eucalyptus stand at three distances (0 m, 50 m, and 100 m) from the border. Half of the stations were netted to prevent bird predation but were accessible to insects. The other half were non-netted and accessible to birds. We conducted six trials, each lasting two weeks, during which we recorded the predation of 504 stations with 5040 pupae. To validate which species predated the pupae, we added video cameras that took RGB videos during the day and IR videos at night in 45 stations and found that in net-free stations, birds preyed in 94.1% of stations in the orchard and 81.8% in the Eucalyptus stand. However, ants predated 70% of the pupae in stations with nets in the orchards and 100% in stations in the Eucalyptus strands. In addition, we found a significant rise in predation by birds as the distance into the orchard increased. Conversely, insect predation declined within the orchard but escalated in the adjacent unmanaged area. These findings suggest that the orchard’s environment negatively affects beneficial insect activity, specifically predatory ants. This study demonstrates that birds can play an essential role in predating insect pests inside the orchard. In addition, we believe that the decreased predation of ants within the orchard was due to intense insecticide use.

## 1. Introduction

The increasing global population and demand for food are putting pressure on agriculture to intensify production, leading to increased use of pesticides. This is causing concerns over negative impacts on human health and the environment, including biodiversity [1,2]. To address this issue, alternative methods for crop production that are less harmful to ecosystems [3,4] and human health are needed. This includes practices such as integrated pest management, crop rotation, and promoting natural predator activities. 

There has been a shift in recent years toward recognizing the positive impacts that wildlife can have on agriculture through the provision of ecosystem services [5,6]. These services can provide valuable benefits to farmers, such as weed control, seed dispersal, pollination, waste removal, disease control, and pest control [7]. By embracing the positive relationship between agriculture and wildlife, it is possible to increase crop production while reducing the need for harmful pesticides and promoting biodiversity. This highlights the importance of considering both the negative and positive impacts of agriculture on the environment and working towards more sustainable practices that benefit both humans and the ecosystem.

Biological control is a strategy for managing pests that emphasizes preserving and promoting beneficial species [8]. This approach leverages natural predators, parasites, and pathogens of pests to achieve effective pest control and minimize reliance on hazardous chemical pesticides. Biological control seeks to maintain a balanced ecosystem and promote long-term pest management while advancing biodiversity conservation by considering the complex interactions between pests, their enemies, and the environment. Compared to conventional pesticide use, which has been shown to have detrimental effects on public health and cause livestock and livestock product losses [9], biological control offers both environmental and economic benefits [10].

Birds play an important role in maintaining a balance in ecosystems by controlling pest populations [11,12], thereby decreasing crop damage and increasing yield [13,14]. To name a few examples, birds have been shown to be used successfully in reducing crop damage by insect pests in coffee plantations [15,16,17], corn [18], and oil palms [19]. 

Insects also play a vital role in regulating populations of other insect pests, making them important components of healthy ecosystems [20]. Many species of predatory insects feed on the eggs and larvae of other insect pests, helping to keep their populations in check. Ladybugs have been utilized as a form of biological pest control since the late 19th century, serving as a natural predator of pests such as aphids, scale insects, and other soft-bodied insects that threaten crops and ornamental plants [21]. Ants sometimes play a crucial role in regulating populations of other insect pests and provide essential ecosystem services through biological control [22]. For example, a meta-analysis of 52 studies on 17 different crops found that ants decrease the abundance of non-honeydew-producing pests and thereby improve crop yield [23].

However, there is still a need for more experimental data to understand the insects’ contribution to ecosystem services and the interplay between functional ecology, community ecology, and biodiversity conservation in the face of ongoing global change [24].

Pest control in apple orchards is crucial for ensuring the health and productivity of the trees and the quality of the fruit produced. Apple orchards are susceptible to a wide range of pests, including insects, diseases, and fungi, which can cause significant damage to the trees and reduce crop yield. To combat these pests, apple growers have traditionally relied on chemical pesticides, which can effectively control pest populations but can also negatively impact the environment and human health. Moreover, chemical pesticide use can also promote pest resistance [25,26], making pest control increasingly difficult. 

The impact of surrounding ecosystems on pest control in apple orchards is significant, as diverse ecosystems can provide a range of resources essential for the survival and reproduction of natural enemies [27,28,29]. For example, a surrounding ecosystem can provide natural enemies with food, habitat, and alternative hosts, enhancing their ability to control pests. Additionally, a diverse surrounding ecosystem can provide refuge for natural enemies from adverse weather conditions and from the direct effects of chemical pesticides, which can reduce their populations in apple orchards. As a result, the surrounding ecosystem can play a crucial role in regulating pest populations and promoting the effectiveness of biological control in apple orchards. 

The coddling moth (*Cydia pomonella*) is a notorious pest of apple orchards worldwide, causing significant economic damage to the fruit industry. The larvae of this moth burrow into the fruit and feed on the seeds, resulting in premature fruit drop and decreased quality of the remaining apples. Controlling coddling moths relies primarily on the use of chemical insecticides, but this approach has several drawbacks, including potential harm to non-target organisms and the development of insecticide resistance. Alternative approaches, such as pheromone-based mating disruption [30], have shown promise in reducing coddling moth populations. 

It has been suggested that insectivorous birds may reduce insect pests in apple orchards [31]. For example, two species of woodpeckers were found to reduce the number of codling moth larvae by 52% in 1949–1956 in Nova Scotia [32]. In another study, silvereyes (*Zosterops lateralis*), a small passerine, were shown to have a functional response when predating on the larvae of the codling moth; the more larvae that were found, the more larvae the birds consumed [33]. There have been attempts to use invertebrates to control the coddling moth but with limited success [34,35]; therefore, looking at other predators is necessary. The widespread use of pesticides in agriculture has been shown to negatively impact the population levels of beneficial predators [36]. Furthermore, the use of pesticides has been shown to have toxic effects on these beneficial species [37], leading to reduced population levels and a decline in their ability to control pest populations effectively [36]. Intense pesticide use can also reduce insect populations serving as a food source for beneficial predators causing a reduction in their populations. This can increase pest pressure and the need for more pesticide application, creating a vicious cycle of pesticide use and its negative environmental impacts.

Our objective was to investigate whether the impact of beneficial predators, such as birds and insects, varies depending on the distance from apple orchards and unmanaged Eucalyptus stands. We hypothesized that predation by birds and insects would be more significant in the unmanaged Eucalyptus stands and at greater distances from the orchard due to increased pesticide use.

## 2. Materials and Methods

The present study was conducted in Moshav Yonatan, Ramat Golan, Israel (32°57′9″ N, 35°49′59″ E), located 548 m above sea level. The study compared predation rates in a commercial apple orchard to those in a nearby unmanaged Eucalyptus stand. The eucalyptus trees were planted in 1960–1970 by the Jewish National Fund as a part of a nationwide campaign to add trees in the country. The eucalyptus trees were also later used to provide shelter for cattle. We used mealworm pupae (*Tenebrio molitor*) to model coddling moth pupae to avoid spreading this pest in the orchards. We used live pupae because previous research has shown that predation on artificial insect pupae models does not always indicate predation on live prey [38]. To ensure that the predation rates of mealworm and codling moth pupae by birds are comparable, we conducted a pilot study and compared the predation rates on feeding stations on 87 trees, each containing five pupae of the two species. We did not find any difference between the predation rate of the two species (Figure S1). Consequently, we decided to utilize mealworm pupae as a substitute for coddling moth pupae. The mealworm larvae were acquired from a nearby retailer and raised on an oatmeal substrate at room temperature until they underwent pupation.

To examine the effect of distance from the apple plot border on mealworm pupae predation, we placed 14 stations with ten pupae at 0 meters, 50 meters, and 100 meters from the plot border inside the apple orchard and in the bordering Eucalyptus stand totaling 84 stations (Figure S2). The 0-m locations were at the border of each habitat, and all stations at each location were placed 3 meters from one another. In each station, ten mealworm pupae were attached to a cardboard platform using glue [39]. To replicate the pupae’s natural environment beneath tree bark, they were positioned between two pieces of cardboard, one of which was affixed to the trunk of an apple or eucalyptus tree at a height of 1.5 meters. To differentiate between predation of pupae by birds and small invertebrates, a netted cage made of plastic mesh with 1 × 1 cm holes was used to enclose half of the feeding stations (every other station) at each location. This prevented birds from accessing the pupae while the other half of the stations were uncovered. The mesh prevented birds but allowed small invertebrates to reach the pupae. After setting up the pupae platforms at each feeding station, we tracked the number of missing pupae at each station after 8 and 15 days and also documented the presence or absence of rip and peck marks on the cardboard as evidence of bird predation. Between 11 May–30 August 2021, we repeated this procedure five times, which yielded a total sample size of 420 stations (14 stations × 6 distances × 5 repetitions). However, due to the elevated rate of predation observed in stations without nets—almost all pupae were predated between days 8 and 15—we conducted an additional experiment on 22 September 2021, wherein we monitored predation at an increased rate of 1, 2, 8, and 15 days after the pupae were placed. During the study, we positioned a total of 5040 pupae.

To confirm the predator of the pupae, we installed Hikvision bullet security cameras as camera traps. These cameras were equipped with a 25-watt solar panel, a solar controller, and a 20 mah battery, allowing continuous recording 24/7 using RGB camera (red, green, and blue) during the day and infrared at night. We placed camera traps at 35 feeding stations without nets and 23 feeding platforms with nets. The cameras were securely installed for the entirety of each repetition, from establishing the feeding stations until their removal. Any camera traps that had lost videos of pupae feeding events or did not provide sufficient footage to detect the predator were excluded from the analysis. Also, one station where a common whitethroat (*Curruca communis*) managed to enter a netted cage with a hole was excluded. 

We used the 2 × 2 χ^2^ test of independence to compare the rates of peck and peel marks between netted and net-free stations. We used the same procedure to compare the predation rates by different bird species. We first tested whether predation intensity (the number of prey eaten per station) at the first five repetitions varied in distance and over time using a generalized linear mixed model (GLMM, IBM SPSS Statistics 25). The number of missing pupae (out of 10) was set as the dependent variable. Given that the data were counts of missing pupae out of 10, the probability distribution was defined as binomial with the logit link function. We analyzed the data from netted and net-free stations separately. The following independent variables were used: Station number (random), Inspection day after the stations were situated (fixed; 8 days and 15 days), Repetition number (fixed), and Location (fixed, 6 locations in the orchard and Eucalyptus stand together at distances 0, 50 and 100 meters from the Eucalyptus stand and apple orchard border). We used pairwise contrasts with the least significant difference (LSD) adjusted significance level (α = 0.05) to test for differences between individual locations. Next, we used a similar model to test predation intensity in a 6th repetition with the independent variables: Station number (random), Inspection day after the stations were situated (fixed; 1, 2, 8, and 15), and Location (same 6 locations as above). Pairwise contrasts were also used, as described above.

## 3. Results

**Figure 1:**
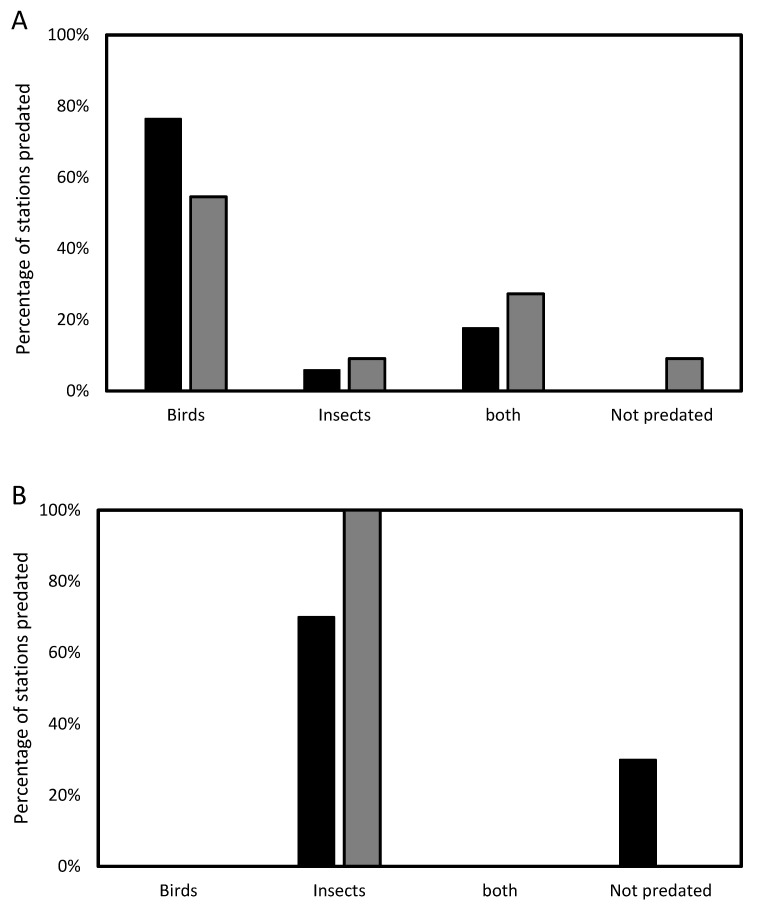
The analysis of camera footage was used to determine the difference in predation between net-free (**A**) and netted (**B**) stations in the orchard (black) and Eucalyptus strand (gray) for birds, insects, a combination of both, and no predation.

The rate of peel and peck marks (signs of predation) 2 weeks after we placed the feeding stations differed between netted and net-free stations. We observed marks in 23% (56 of 242) of net-free stations and 3% (7 of 203) of netted stations (χ^2^ test, *p* < 0.001). Cameras were installed in 44 stations, and recorded predations were discovered in 90.9% of the stations (Table 1). Table 1 summarizes the data obtained from the camera traps in all six experimental repetitions. The camera traps captured predation by two bird species, unidentified ant species, and two instances of predation by other unidentified insects (Table 1). In some stations, both birds and ants were observed predating in the same station. However, there were no cases of more than one bird species at the same station and session. Birds carried out 94.1% of predation in net-free stations in orchards and in Eucalyptus stands, birds did 81.8% of predation in net-free stations. Conversely, in netted stations, insect predation was observed in 70% of orchard predation cases and in 100% of the cases in the Eucalyptus stands (Figure 1). The white-spectacled bulbul (*Pycnonotus xanthopygos*) and great tits both were found to prey on 27.3% of the stations, while ants (species unknown) preyed on 47.7% of them (see Supplementary Materials Videos S1–S3).

In 23 out of 28 net-free traps with cameras, pupae were preyed on by two bird species- bulbuls and Great tits. However, ants were the sole predator in only one station without a net in the orchard and two stations in the Eucalyptus stand (Table 1). Additionally, there was no predation of the pupae in one of the net-free stations located in the Eucalyptus stand. Interestingly, bird species visitation rates differed between the two habitats: in the orchard, bird predation was done predominantly by bulbuls (69%), and in the Eucalyptus stand, it was biased towards great tits (89%) (χ^2^ test, *p* = 0.027). 

**Figure 2:**
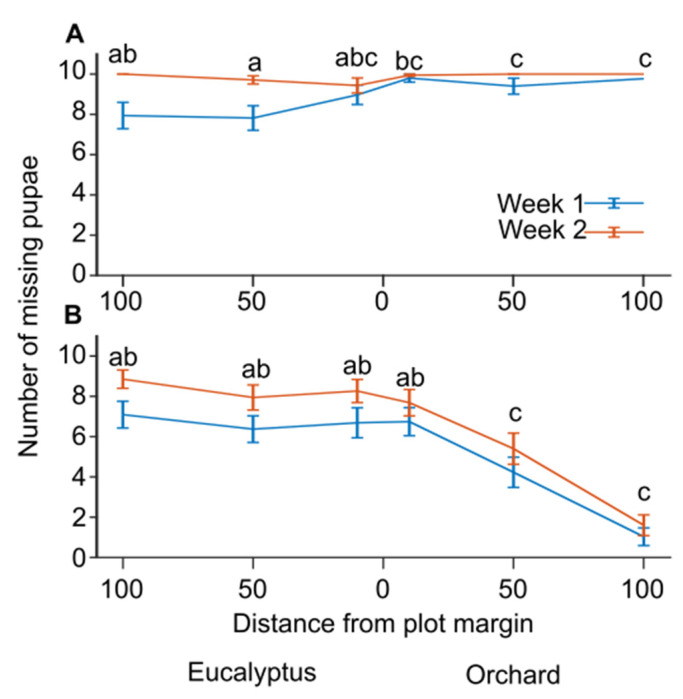
The relation between pupae predation and habitat after 1 or 2 weeks (8 or 15 days). The plot shows the cumulative numbers of missing pupae (out of 10) at feeding stations (mean ± s.e.m, N = 30 to 35) placed in an orchard or the neighboring Eucalyptus stand at distances of 0, 50, or 100 meters from the border between the two habitats. The red and blue lines indicate missing pupae numbers 8 or 15 days after the feeding stations were placed. (**A**) net-free station; (**B**) netted stations. The letters above the graphs in (**A**,**B**) indicate significant differences between locations resulting from pairwise contrasts with an LSD-adjusted significance level α = 0.05, following the GLMM procedure.

We compared the number of missing pupae in the feeding stations 8 or 15 days after the cardboards with pupae lures were placed at the stations for a pooled sample of the first five repetitions (sample sizes were between 30–35 stations per Location for netted and net-free stations; Figure 2). Predation in the net-free stations was higher in the orchard than in the Eucalyptus stand, specifically 50 and 100 meters inside the orchard compared to the matching distances inside the neighboring stand (Figure 2A; generalized linear mixed model with a binomial distribution and logit link function, F_9,394_ = 6.5, *p* < 0.001; pairwise contrasts with LSD adjusted significance level α = 0.05). However, almost all the pupae in the net-free feeding stations were missing by the 2nd week. In the GLMM, Inspection day and Repetition effects were both significant; *p* < 0.001 and *p* = 0.001, respectively. Predation in the netted stations also varied significantly between the orchard and the Eucalyptus stand, but with an opposite trend (Figure 2B; generalized linear mixed model with a binomial distribution and logit link function, F_10,402_ = 11.1, *p* < 0.001; pairwise contrasts with LSD adjusted significance level α = 0.05). Specifically, predation inside the orchard decreased 50 meters and 100 meters from plot margins. In the GLMM, Inspection day and Repetition were also significant, *p* = 0.001 and *p* < 0.001, respectively. These results suggest that predation rates in stations with nets were higher in the Eucalyptus stand, whereas predation in the net-free stations was higher in the orchard.

**Figure 3:**
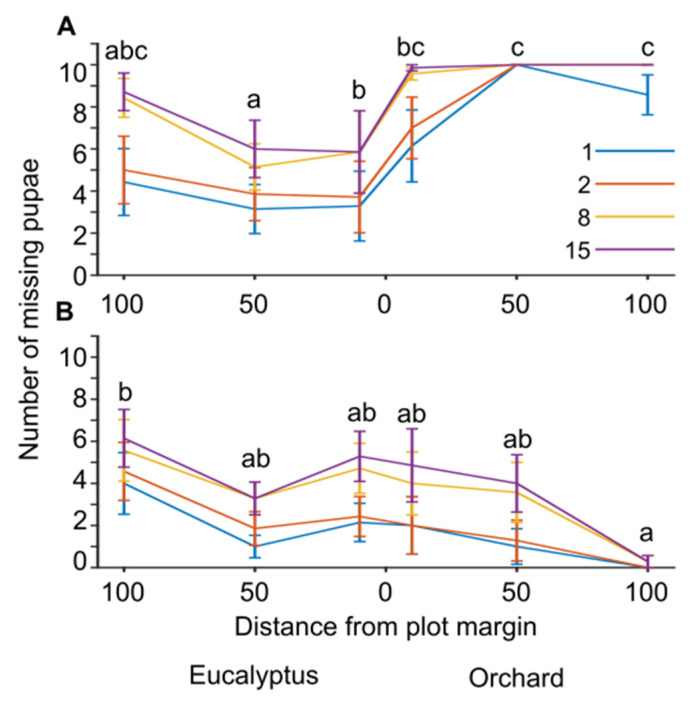
The relation between moth pupae predation and habitat after 1–15 days. The plot shows the cumulative numbers of missing pupae (out of 10) at feeding stations (mean ± s.e.m, N = 7) placed in the apple orchard or neighboring Eucalyptus stand at distances of 0, 50, or 100 meters from the border between the two habitats. The colored lines indicate missing pupae numbers 1, 2, 8, or 15 days after the feeding station was placed. (**A**) net-free station; (**B**) netted stations. The letters above the graphs in (**A**,**B**) indicate significant differences between locations resulting from pairwise contrasts with an LSD-adjusted significance level α = 0.05, following the GLMM procedure.

Given the high predation rates and that most pupae were eaten within two weeks in the net-free stations, we repeated the observations one more time and increased monitoring frequency to 1, 2, 8, and 15 days after the feeding stations were placed (sample sizes were N = 7 stations per Location; Figure 3). We found that feeding rates increased in net-free stations at 50 and 100 meters inside the orchard and decreased both in the orchard margins and in the eucalyptus stand (Figure 3A; generalized linear mixed model with a binomial distribution and logit link function, F_8,159_ = 20.1, *p* < 0.001; pairwise contrasts with LSD adjusted significance level α = 0.05). In the GLMM, predation varied with Inspection day (*p* < 0.001). In netted stations, predation rates were highest at 100 meters in the Eucalyptus stand and lowest deep inside the orchard at 100 meters from orchard margins (Figure 3B; generalized linear mixed model with a binomial distribution and logit link function, F_8,159_ = 15.9, *p* < 0.001; pairwise contrasts with LSD adjusted significance level α = 0.05). In the GLMM, predation varied during the Inspection day (*p* < 0.001). These results suggest that bird predation significantly increased with increasing distance into the orchard. On the other hand, insect predation decreased in the orchard and increased in the nearby unmanaged Eucalyptus stand.

## 4. Discussion

This study aimed to compare the predation rates of mealworm pupae as models for insect pests at different distances within and between an apple orchard, and Eucalyptus stand. Initially, we compared the predation rates of coddling moth and mealworm pupae. We found that both were similarly predated, with higher predation observed in feeding stations without nets than those with nets. We then examined whether predation varied in netted and net-free feeding stations at different distances in orchards with conventional pesticide use and disturbances and in a Eucalyptus stand with lower disturbance and pesticide use. Our hypothesis that more predation in the Eucalyptus stand was only supported in netted feeding stations. This is likely due to the increased abundance of ants in this unmanaged habitat. Interestingly, the predation rate by ants dropped significantly from 1–100 meters within the insecticide treated orchard. Surprisingly, in the net-free stations, predation was higher in the apple orchard, and the predation rate increased with distance into the orchard. Furthermore, the difference in predation was evident in the first two days after the feeding stations were implemented. These results suggest that the orchard environment supports increased bird activity while the nearby stand habitat is associated with increased ant activity. 

We found that insect predation, primarily attributed to ants, decreased with increasing distance within the orchards. We suspect this is due to the increased pesticide use in the orchard, which can affect ant populations by killing them directly or reducing the availability of invertebrate prey. In a related study, researchers investigated the impact of pesticide use on the population sizes of the beneficial predator *Notonomus gravis* in agricultural fields [40]. They found that as the distance increased into the crop fields, the population sizes of *Notonomus gravis* declined. This decline was attributed to the increased use of pesticides, which can directly or indirectly affect the predator’s survival or reproductive success. However, in neighboring remnant grasslands, the population sizes of *Notonomus gravis* increased, possibly due to the reduced pesticide exposure or the presence of alternative prey or suitable habitats. These studies suggest that pesticide use can have complex and varied impacts on insect populations in agricultural fields, with potential consequences for pest control and ecosystem health. Ants have been identified as effective biological pest control agents [41]. In our study, we could not identify the species of ants or other insects involved in predation, which could be significant in understanding their role in pest control. Future research must explore alternative pest control methods that minimize harm to beneficial insects while effectively controlling pests in agricultural settings.

We also observed a difference in predation between stations that were exposed to birds and those that were not, with higher predation pressure observed in stations that were accessible to bird predators (without nets) compared to those that were not (with nets). Compared to the netted stations, in most unnetted stations, all pupae were predated upon after 15 days. Many pupae were preyed upon 8–15 days after being placed in the stations, indicating they remained attractive to the predators. It is worth noting that bird predation was more prevalent in the orchards than in the Eucalyptus stand, potentially attributed to the greater abundance of bulbuls, as observed through our video analyses. Birds were the main predators of the pupae in net-free stations in the apple orchard and the Eucalyptus stand. One hypothesis explaining the predation difference may be that fewer herbivorous insects exist in the Eucalyptus trees and, therefore, fewer predators. However, this seems unlikely, given that the orchard is treated with pesticides. Therefore the Eucalyptus stand may be less attractive to birds for other yet unknown reasons, which need to be further studied. A previous study showed that excluding bird predation in net-covered trees significantly increased codling moth abundance and fruit damage [42]. In addition, insectivorous birds can induce top-down control of insect pests, benefiting the plants that herbivores would otherwise consume [43]. It should be noted, however, that while great tits predominantly feed on insects, bulbuls can consume fruits and harm crops. As a result, farmers could profit from boosting bird populations [14,42,44]; however, this is contingent on ensuring that the avian species in question do not harm crops. Thus, it is crucial to assess whether these birds contribute to ecological benefits that outweigh any potential damages they may cause to crops.

Our pupae application method may bias predation rates compared to natural codling moth predation. Upon completing their feeding phase in the apple fruit, codling moth larvae transition to the tree trunk, locate crevices in the bark to hide and protect their cocoons, and eventually undergo pupation [45]. During this phase, great tits and other bird species can detect and consume the concealed cocoons within the tree bark. In our experiment, we attached mealworm pupae to the tree trunk in cardboard pockets to model the hidden codling moth pupae. Using this method, it may be easier for bird predators to locate the pupae lures compared to the naturally hidden pupae. Therefore the total rate of predation we found may not reflect the actual rate of codling moth predation. In addition, we did not investigate other stages of the pest life cycle, which could be subject to different predation pressures. However, given that the same method was used in all feeding stations, we believe the differences reflect actual differences in predation pressure. 

The use of pesticides in agriculture can have detrimental effects on wildlife, especially bird populations. Studies have consistently shown that bird abundance [46], species richness, and diversity are often lower in conventional orchards where pesticides are commonly used compared to organic orchards where pesticides are either not used or are used in a more limited manner. The current findings highlight the importance of considering the impacts of pest control strategies on wildlife and the potential benefits of using more sustainable practices, such as organic agriculture, for both crop production and wildlife conservation.

Farmers may profit from increasing beneficial predators. Incorporating nest boxes for birds is a potential strategy for augmenting predatorial bird populations, as studies have demonstrated that this can heighten predator pressure, ultimately resulting in reduced pest incidence [14]. Since the occupation of nest boxes is related to habitat quality, studies need to determine whether habitat restoration/diversification could increase ecosystem services and reduce the need for pesticides. Among the most promising strategies for controlling coddling moths is using great tits [47,48]. The addition of nest boxes increased predation rates on caterpillars by 32% in Spain [49]. In one study, predation of coddling moth larvae by great tits increased apple yield from 4.7 to 7.8 kg per tree [48]. In another experiment, only 8% of coddling moth larvae reached adulthood in areas with predation by great tits compared to 48% in predator-free controls [47].

Furthermore, it was estimated that 44% of the larvae matured in apples failed to build cocoons on the trees, and 47% were taken by tits [47,48]. Even though great tits have been observed using nest boxes in Israeli villages [50,51], they have not been found to occupy nest boxes in orchards, which could be attributed to the rise in pesticide usage (Charter, unpublished data). Additional research is necessary to determine if nest boxes can boost great tit populations in orchards where insecticides are prevalent and to assess the impact of these insecticides on the birds in toxicology studies.

Overall, the study provides valuable insights into the differences in predation between an orchard and a neighboring unmanaged habitat and provides surprising results on the high bird predation rates in the managed orchard. This highlights the need for further research to understand the role of different predators in pest control fully. 

## 5. Conclusions

In conclusion, ecosystem services are crucial in pest control, providing natural enemies with the resources they need to control pests effectively. By preserving and enhancing the surrounding ecosystems in apple orchards, growers can promote the populations of natural enemies and reduce pest damage, thereby enhancing the health and productivity of their orchards. These findings highlight the importance of conserving and restoring ecosystems to provide ecosystem services and maintain sustainable pest management in apple orchards.

## Figures and Tables

**Table 1:** The number of cameras placed at feeding stations where different predators were observed.

Net	Location	Bulbul	Great Tit	Ants	Other Insects	Not Predation	Total Number of Traps
net-free	Orchard	11	5	4	0	0	17
Netted	Orchard	0	0	7	2	3	10
net-free	Eucalyptus	1	7	4	0	1	11
Netted	Eucalyptus	0	0	6	0	0	6

## Data Availability

Not available.

## Supplementary Materials

The following supporting information can be downloaded at: https://www.mdpi.com/article/10.3390/ani13111785/s1, Figure S1: Comparison between the predation rate of coddling moths and mealworms; Figure S2: Experimental design of the study site.; Video S1: Example of a white-spectacled bulbul eating at a station. Video S2: Example of a great tit eating at a station. Video S3: Example of ants eating at a station.
