# Supplementary material for: Comparing Insect Predation by Birds and Insects in an Apple Orchard and Neighboring Unmanaged Habitat: Implications for Ecosystem Services
Source: Animals (Basel). 2023 May 27;13(11):1785. doi: 10.3390/ani13111785 (PMC10252115; doi:10.3390/ani13111785)
Supplement: Supplementary file 1 [file animals-13-01785-s001.zip › animals-2349139-supplementary.pdf]

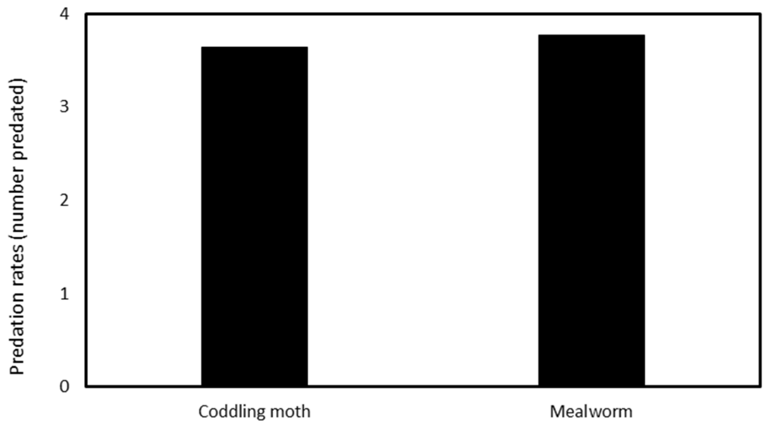

Figure S1. Comparison between the predation rate of coddling moths and mealworms. To ensure that the predation rates of mealworm and codling moth pupae by birds are comparable, we conducted a pilot study and compared the predation rates in feeding stations on 87 trees, each containing five pupae of the two species. The predation rates of the two were similar (Wilcoxon Signed Ranks Test; Z= 1.33, P= 0.18).

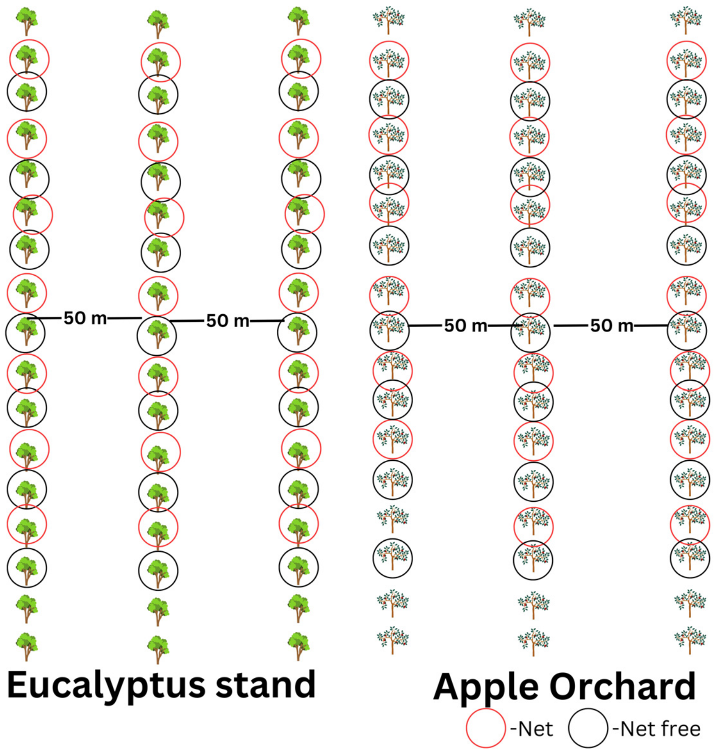

Figure S2. Experimental design of the study site.

Video S1. Example of a white-spectacled bulbul eating at a station: https://youtu.be/wExQagFhV1Q

Video S2. Example of a great tit eating at a station: https://youtu.be/qPQz1zUBf7Y

Video S3. Example of ants eating at a station: https://youtu.be/Fl1E10A8kAE
